# Supplementary figures and images for: Plastid-Targeted Cyanobacterial Flavodiiron Proteins Maintain Carbohydrate Turnover and Enhance Drought Stress Tolerance in Barley
Source: Front Plant Sci. 2021 Jan 13;11:613731. doi: 10.3389/fpls.2020.613731 (PMC7838373; doi:10.3389/fpls.2020.613731)

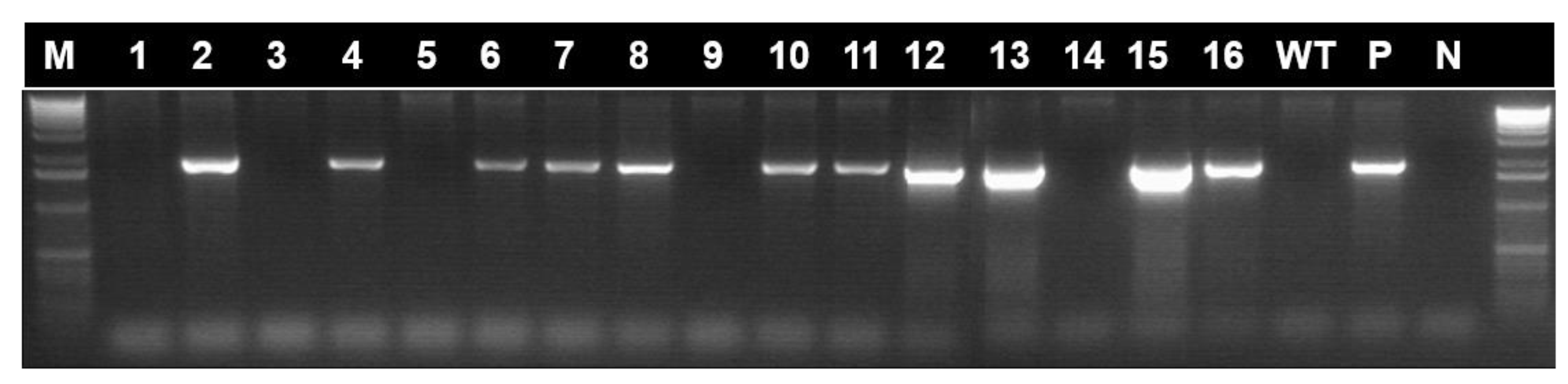

Supplement: Supplementary Figure 1 — A representative segregation analysis of the Flv1 transgene in a set of barley T2 individuals (lanes 1-16), as determined by PCR amplification. The selection of single-locus transgenic plants was made based on a monogenic (3:1) ratio for both Flv1 and Flv3. M: 1 kbp DNA ladder, WT: wild-type, P: empty plasmid control, N: no-template negative control. The size of the target amplicon was 1.8 kbp. Plants lacking the Flv1 amplicon (i.e., lanes 1, 3, 5, 9, 14) were used to produce azygous individuals. [file Image_1.TIF]

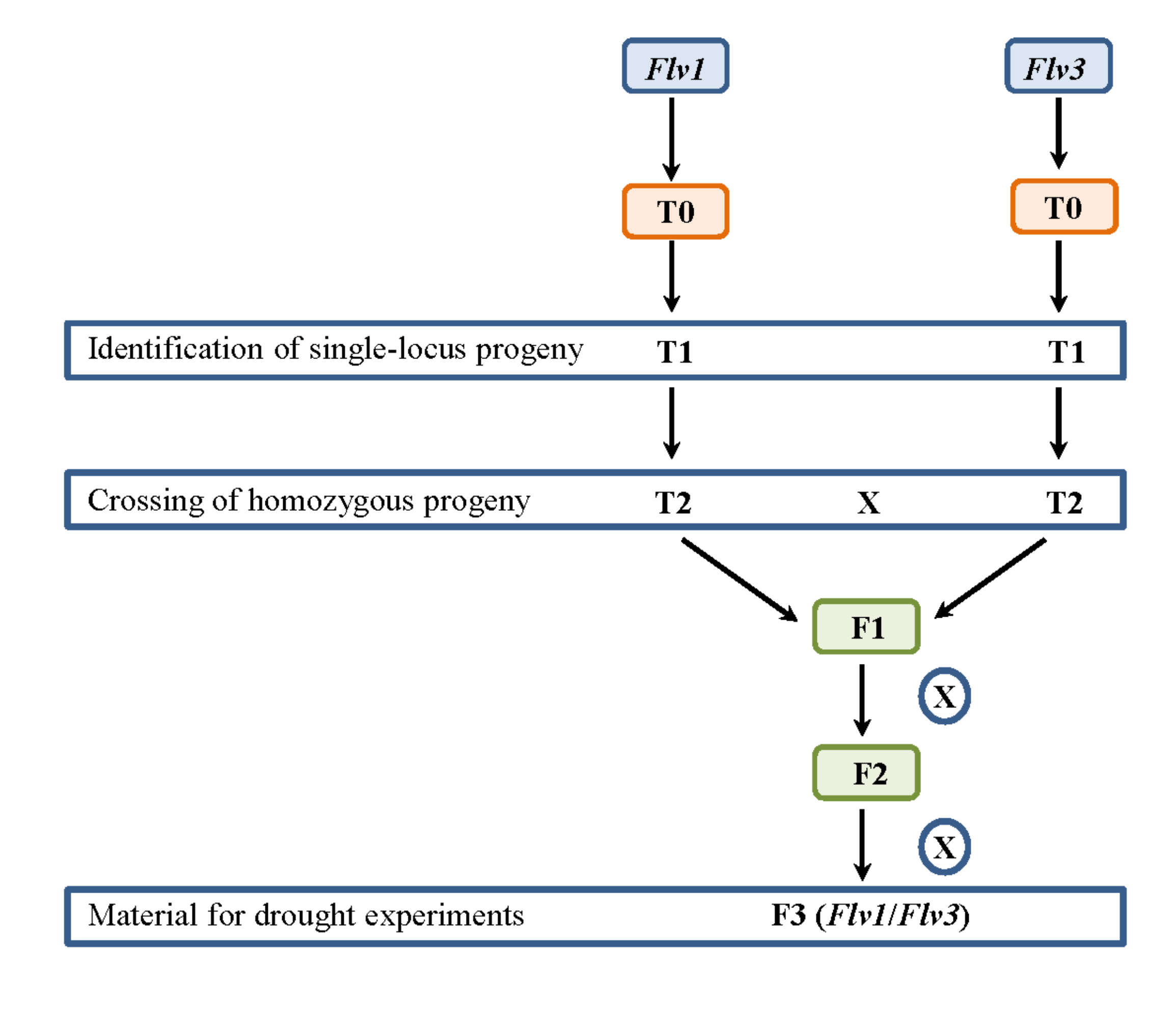

Supplement: Supplementary Figure 2 — A model describing the steps for producing double-homozygous plants harboring Flv1/Flv3 to conduct drought experiments. [file Image_2.TIF]

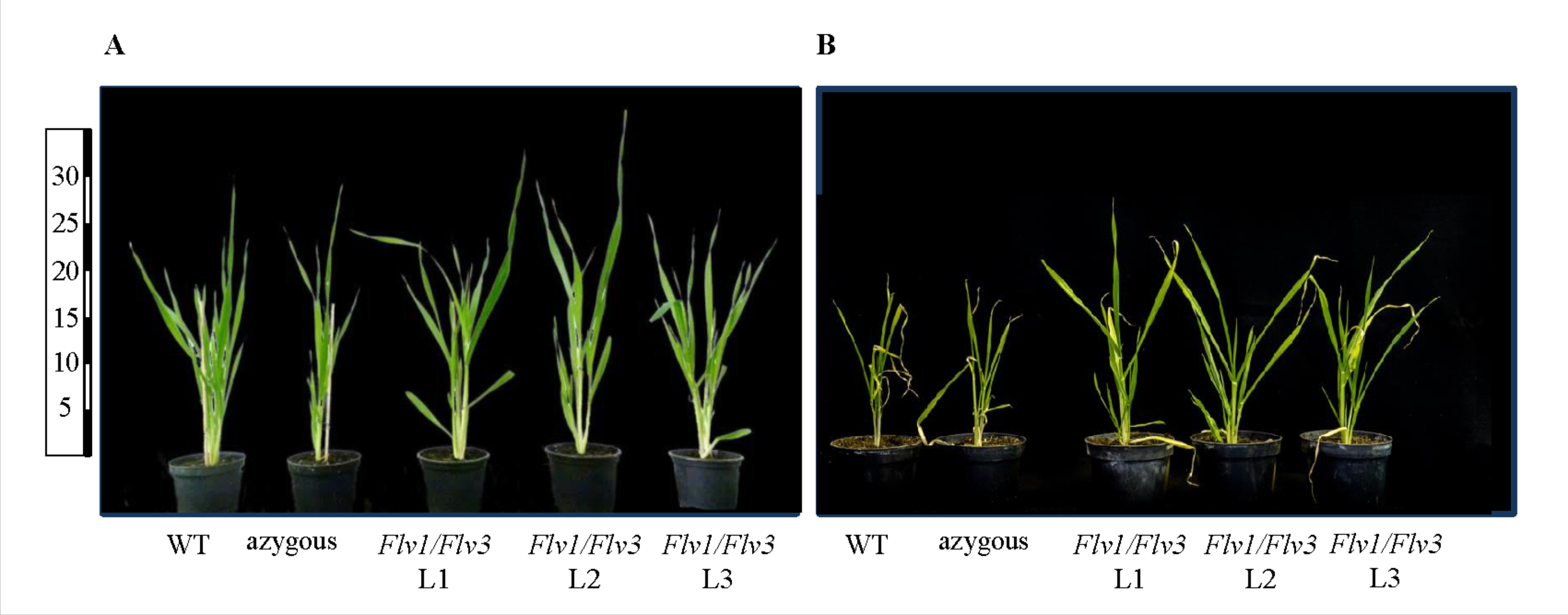

Supplement: Supplementary Figure 3 — The appearance of typical barley plants heterologously expressing Flv1/Flv3 genes at the seedling stage under ambient (A) and drought-stressed (B) conditions. Lines L1-L3 harbor both Flv1 and Flv3 genes. Images captured seven days after rewatering from a soil maintained at 10-12% FC for 5 days. Growth performance of WT, azygous and transgenic plants in ambient condition (A). Seven days after re-watering, WT barley plants exposed to severe drought exhibited retarded growth and leaf wilting, while leaves of the three transgenic lines retained turgor (albeit turning slightly yellowish). Numerals on the left indicate height in cm. [file Image_3.TIF]

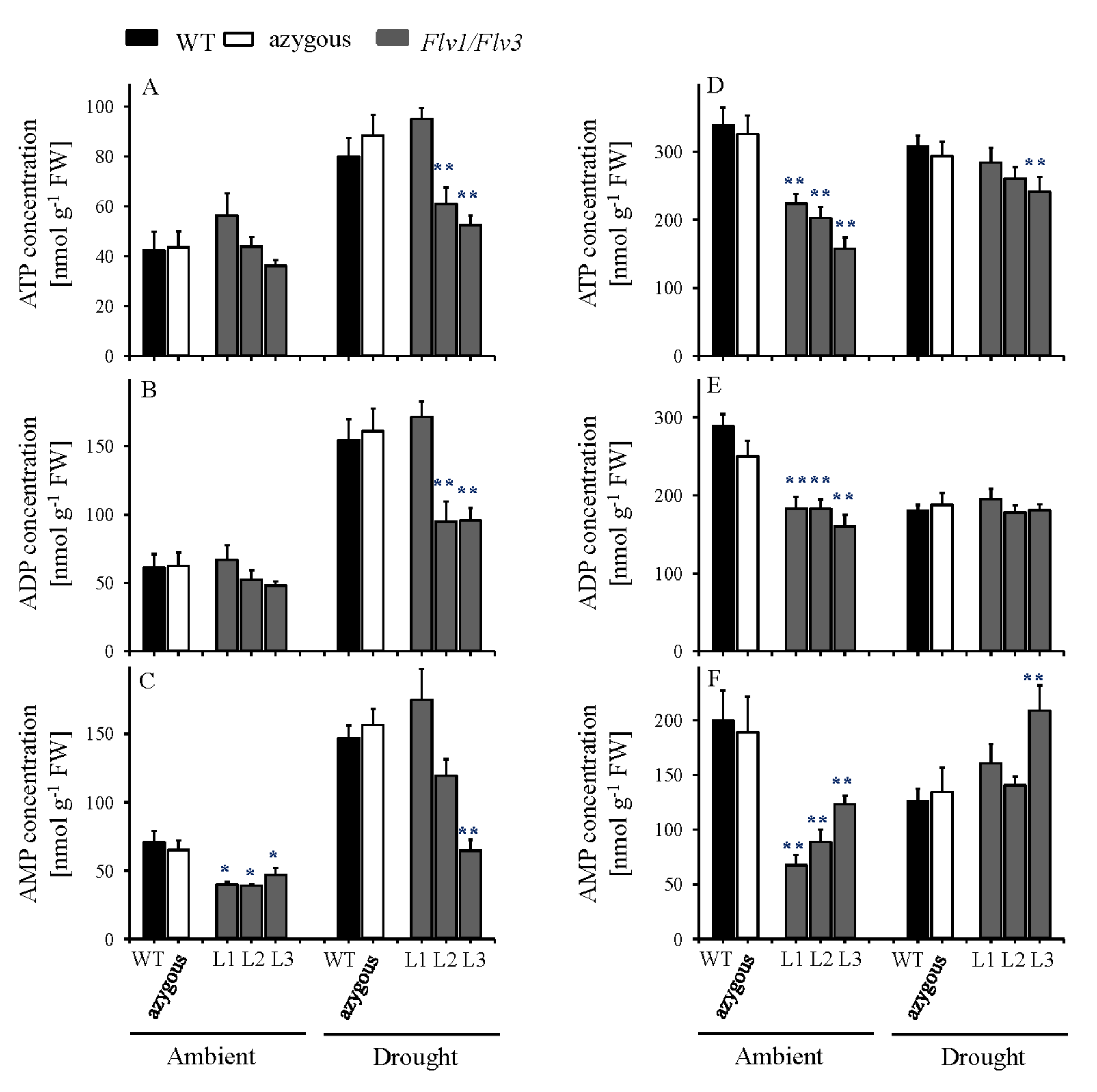

Supplement: Supplementary Figure 4 — Influence of heterologously expressing Flv genes on adenosine nucleotides in the flag leaves of barley plants grown either under ambient conditions or exposed to drought stress at the seedling stage (A–C) and the reproductive stage (D–F). Adenine nucleotide levels were measured in the same samples used for carbohydrate determinations (Figure 5). (A,D) ATP, (B,E) ADP, (C,F) AMP. Lines L1-L3 harbor both Flv1 and Flv3 genes. Data are shown as means ± SE (n = 5-6). ∗∗; ∗: means differed significantly (P ≤ 0.01 or P ≤ 0.05, respectively) from those of non-transgenic plants. FW, fresh weight. [file Image_4.TIF]

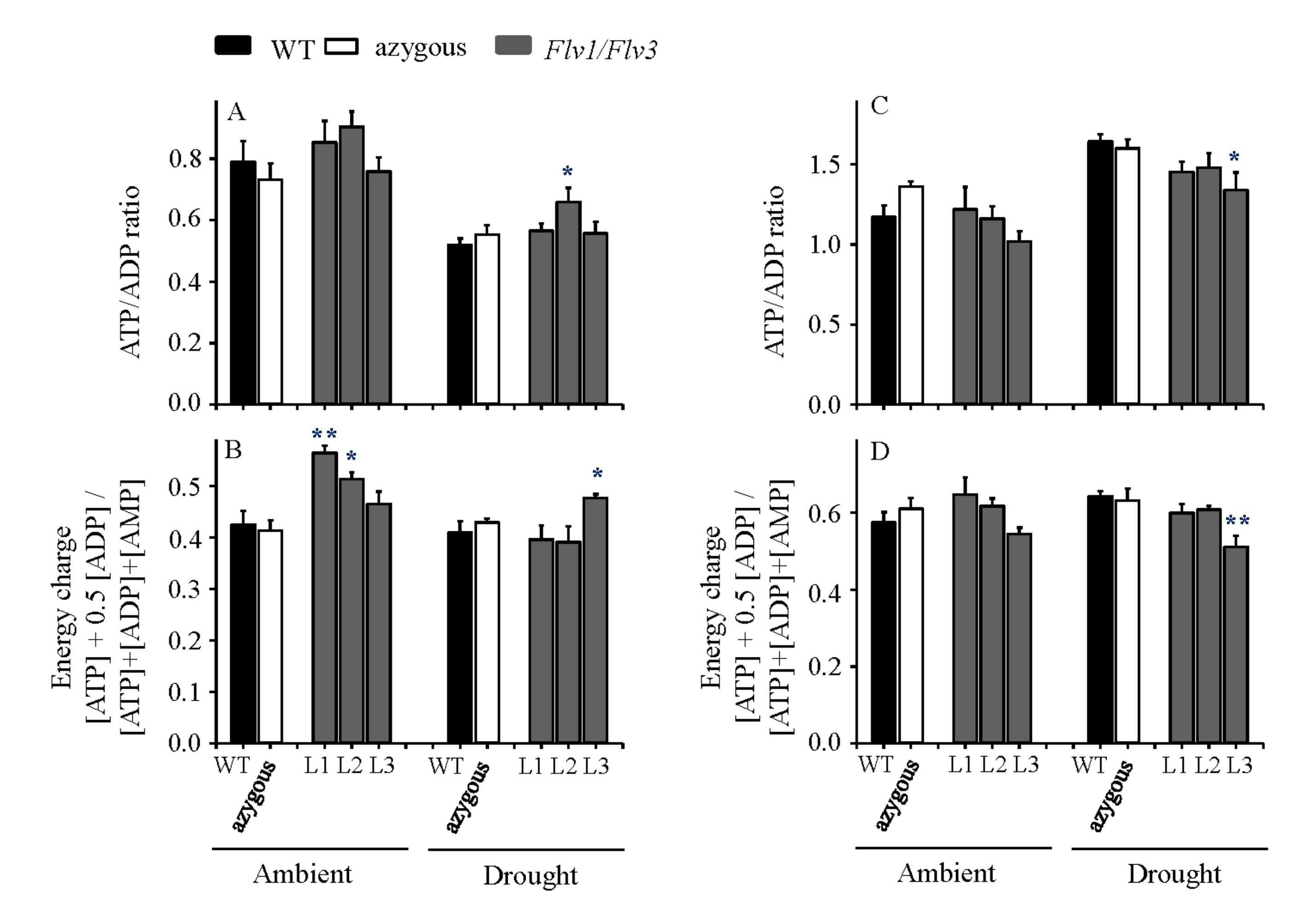

Supplement: Supplementary Figure 5 — Effect of heterologously expressing Flv genes on the energy status of flag leaves of barley plants grown either under ambient conditions or exposed to drought stress at the seedling stage (A,B) and the reproductive stage (C,D). (A,C) ATP to ADP ratio, (B,D) energy charge. Lines L1-L3 co-express Flv1 and Flv3 genes. Data are shown as means ± SE (n = 5-6). ∗∗; ∗: means differed significantly (P ≤ 0.01 or P ≤ 0.05, respectively) from the performance of non-transgenic plants. [file Image_5.TIF]
